# Anomalous refraction and reflection characteristics of bend V-shaped antenna metasurfaces

**DOI:** 10.1038/s41598-019-43138-1

**Published:** 2019-04-30

**Authors:** Yanqiang Xie, Chang Yang, Yun Wang, Yun Shen, Xiaohua Deng, Binbin Zhou, Juncheng Cao

**Affiliations:** 1 Department of Physics, Nanchang university, Nanchang, 330031 China; 2 Institute of Space Science and Technology, Nanchang university, Nanchang, 330031 China; 3 DTU Fotonik, Technical University of Denmark, Building 343, DK-2800 Kgs Lyngby, Denmark; 4 Key Laboratory of Terahertz Solid-State Technology, Shanghai Institute of Microsystem and Information Technology, Chinese Academy of Sciences, Shanghai, 200050 China

**Keywords:** Physics, Optics and photonics

## Abstract

Stabilization issue of anomalous refraction and reflection in V-shaped antenna metasurfaces are investigated. Specifically, when a V-shaped metasurface is artificially tilted, the induced refraction and reflection are theoretically analyzed. Detailed numerical and experimental study is then performed for the upward and downward bending metasurfaces. Our results show that although the anomalous reflection is sensitive to the deformation of metasurface geometry; the anomalous refraction is, surprisingly, barely affected by relatively small-angle tilting and able to support perfect beam orienting. Since in real-world applications, the optical objects are often affected by multiple uncertain factors, such as deformation, vibration, non-standard surface, non-perfect planar, etc., the stabilization of optical functionality has therefore been a long-standing design challenge for optical engineering. We believe our findings can shed new light on this stability issue.

## Introduction

Metasurface has attracted a lot of attentions due to its gorgeous performances and ultrathin thickness compared to conventional bulk optical components^1–3^. It is composed of 2D nanostructure arrays with gradient phase change, and can arbitrarily manipulate magnitude, phase, and polarization of electromagnetic waves at a sub-wavelength scale along propagation direction. So far, metasurfaces of V-shaped antennas, C-shaped antennas and rectangular patches have been reported to artificially control the characteristics of waves^4–6^. In specific, when a polarized wave is incident on the metasurface antennas, both antenna modes can be excited but with substantially different amplitude and phase caused by their distinctive resonance conditions, then anomalous cross-polarized scattered light are produced. Based on these exotic properties, various metasurface-based optical devices including flat lens^7–10^, beam reflectors^11,12^, wave plates^13,14^, vortex generators^15–17^, meta-deflectors^18^, holograms^19–21^ and other novel, high-performance versatile photonic metadevices^22,23^ have been studied. Nonetheless, the optical characteristics are usually correlated with the geometry of the designed object, for example, cylinders lead light to a line, spheres result in a point and arbitrarily shaped objects introduce optical aberrations. As in actual operation the geometry is affected by many uncertain factors including deformation, vibration, non-standard surfaces, non-perfectly planar, etc., all of which increase the uncertainty of light manipulation in phase, amplitude and polarization^22,23^, stabilization of the optical functionalities has been a long-standing design challenges for optical engineering.

In this paper, the stabilization of anomalous refraction and reflection characteristics in a bended V-shaped antenna metasurface are investigated. Theoretical and experimental results show that the anomalous reflection is sensitive to deformation of the metasurface geometry. In contrast, the anomalous refraction is, surprisingly, barely affected by relatively small-angle tilting or bending and able to support perfect beam orienting.

## Results

### Operation principle and analysis

**Figure 1:**
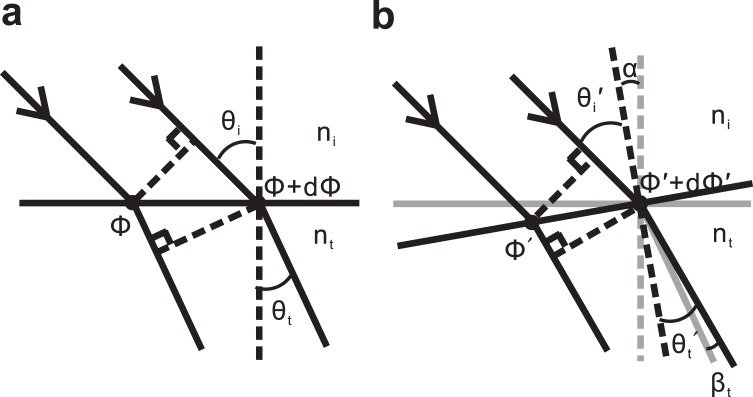
Light propagation in metasurface. (**a**) Schematic used to derive the generalized Snell’s law of refraction. (**b**) Refraction path considering the initial interface of (**a**) is rotated *α* angle.

As is known, a metasurface consists of arrays of plasmonic antennas with linear gradient phase can generate anomalous reflection and refraction which is in agreement with generalized Snell’s laws derived from Fermat’s principle. When a light is incident on the interface of Fig. 1(a) at angle *θ*_i_, due to the Fermat’s principle, phase difference between the two light paths should be zero and satisfy *k*_0_*n*_*i*_ sin (*θ*_i_)*dx* + (*ϕ* + *dϕ*) = *k*_0_*n*_*t*_ sin (*θ*_t_)*dx* + *ϕ*. In which, *θ*_t_ is angle of refraction, *dϕ* and *ϕ* + *dϕ* are severally the phase discontinuities at locations where the two paths cross the interface, dx is the distance between the crossing points. n_i_ and n_t_ are refractive indices, *k*_0_ is vacuum wave vector. Then, from above we can get the generalized Snell’s law of refraction^1^

1 $${n}_{t}\,\sin \,({\theta }_{t})-{n}_{i}\,\sin \,({\theta }_{i})=\frac{{\lambda }_{0}}{2\pi }\frac{d\phi }{dx}$$

Similar to the refraction, it has

2 $$\sin \,({\theta }_{r})-\,\sin \,({\theta }_{i})=\frac{{\lambda }_{0}}{2\pi {n}_{i}}\frac{d\phi }{dx}$$

where *θ*_*r*_ is the reflection angle.

In actual operation, uncertain factors such as deformation, vibration, non-standard surfaces, non-perfectly planar, etc., may affect the geometry of an optical object and thus the stabilization of its functionalities. To investigate the stabilization of the metasurface’s optical characteristics, we first analyze the impact on the anomalous refraction and reflection when the metasurface is artificially tilted. Considering the initial interface of Fig. 1(a) is rotated *α* angle and become tilted interface of Fig. 1(b), where the original refraction path and normal vector for the initial interface are marked by gray lines. In Fig. 1(b), ${\theta ^{\prime} }_{i}$ and ${\theta ^{\prime} }_{t}$ are severally the new angle of incidence and refraction, *dϕ*′ and *ϕ*′ + *dϕ*′ are the phase discontinuities at the locations where the two light paths cross tilted interface, respectively. *β*_*t*_ is the angle difference between refraction for the initial and tilted interfaces, and ${\theta ^{\prime} }_{i}={\theta }_{i}-\alpha$, ${\theta ^{\prime} }_{t}={\theta }_{t}+{\beta }_{t}-\alpha$. According to Snell’s law^21^, it has:

3 $${n}_{t}\,\sin \,({\theta }_{t}+{\beta }_{t}-\alpha )-{n}_{i}\,\sin \,({\theta }_{i}-\alpha )=\frac{{\lambda }_{0}}{2\pi }\frac{d\phi ^{\prime} }{dx^{\prime} }$$

We note that when phase gradient along the interface are designed to be constant, $\frac{d\varphi ^{\prime} }{dx^{\prime} }$ in Eq. (3) will be equal to $\frac{d\varphi }{dx}$ in Eq. (1). Especially, if the metasurface is thin enough, it can be treated as an interface and *n*_*t*_ ≈ *n*_*i*_ in Eq. (3) can be provided. Under these conditions, for small *α*, we can get *β*_*t*_ ≈ 0 due to Eqs (1, 3). This implies that a small tilting rotating angle of the metasurface can almost have no impact on the propagation direction of its anomalous refractions.

Similarly, for the reflection, it has ${\theta ^{\prime} }_{i}={\theta }_{i}-\alpha$, ${\theta ^{\prime} }_{r}={\theta }_{t}+\alpha -{\beta }_{r}$, and

4 $$\sin \,({\theta }_{r}+\alpha -{\beta }_{r})-\,\sin \,({\theta }_{i}-\alpha )=\frac{{\lambda }_{0}}{2\pi {n}_{i}}\frac{d\phi ^{\prime} }{dx^{\prime} }$$

While *β*_*r*_ is the angle difference between reflections for initial and tilted interfaces. From Eqs (2, 4) we can know even *α* is small, changed angle *β*_*r*_ ≈ 2*α* still can be produced.

Evidently, the above results demonstrate that the anomalous reflection is sensitive to tilting rotations of the object, however, the anomalous refraction direction only be insignificantly affected by the tilting and lead to perfect stabilization of light propagation direction. Such stabilization can be attributed to the peculiar physical structure and mechanism of metasurface for anomalous refraction. That is, in general refraction system, it has *n*_*t*_ sin (*θ*_*t*_) = *n*_*i*_ sin (*θ*_*i*_), and the refraction is caused by the difference of *n*_*t*_ and *n*_*i*_, a tilting angle *α* will result in new incident *θ*_*i*_ − *α* and refraction *θ*_*t*_ + *β*_*t*_ − *α*, and the refraction requirement of *n*_*i*_ ≠ *n*_*t*_ will make direction changed angle *β*_*t*_ impossible be zero. In metasurface, the refraction is mainly caused by the $\frac{{\lambda }_{0}}{2\pi }\frac{d\phi ^{\prime} }{dx^{\prime} }$ (Eq. (3)). Especially when it is thin enough and treated as interface, approximate *n*_*i*_ = *n*_*t*_ make direction changed angle *β*_*t*_ ≈ 0 be possible for small *α* due to Eqs (1,3). While in system such as grating and mirror, the direction control of the light beam is primarily dominated by reflection, which is sensitive to the tilting *α*. Comparatively, only the anomalous refraction by metasurface can offer direction changed angle *β*_*t*_ ≈ 0 for *α* tilting. This has important significance to stabilize beam orienting under various conditions such as deformation, vibration, non-standard surfaces, non-perfectly planar in actual operation, which are bound up with the arrangements of various tilting.

### Designed procedure of metasurfaces

**Figure 2:**
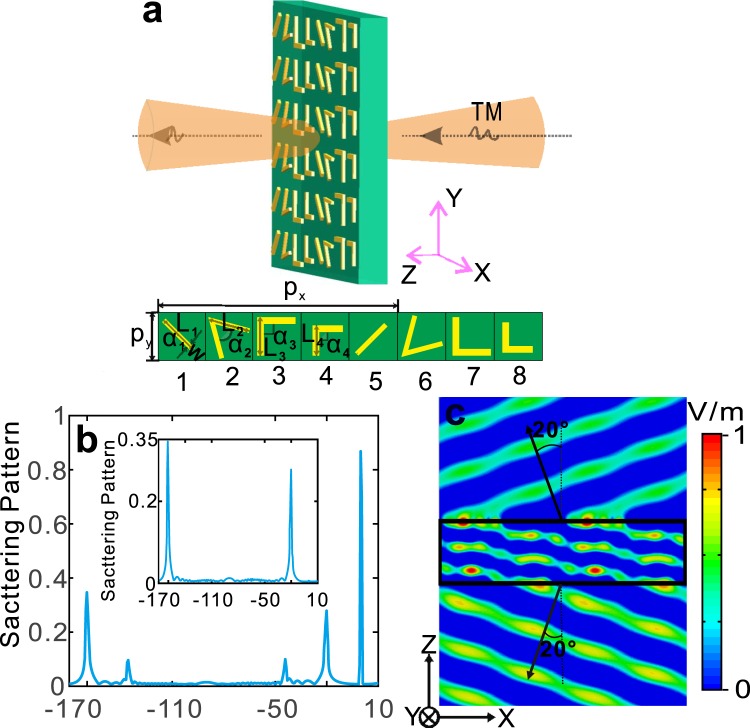
Designed flat V-shaped metasurface and its characteristics. (**a**) Schematic of proposed V-shaped metasurface. (**b**) Total scattering pattern when TM wave (electric field along y direction) is incident on the metasurface; scattering pattern of cross-polarized Ex is shown in the inset. (**c**) Field distribution of cross-polarized Ex along propagation direction (x-z plane).

Specifically, the schematic of the V-shaped antenna metasurface is shown in Fig. 2(a). In which, an array of super-unit-cells arranged periodically is fabricated on flexible substrate polyimide (PI) and the super-cell includes eight anisotropic V-shaped resonating units. Resonant units with gradient phase spacing are arranged in a linear manner, this super-unit-cells can cover a phase of 0 to 2π and can achieve anomalous refraction and reflection with cross-polarization, obeying generalized Snell’s law^21^. Here we take 4.3 THz (corresponding to wavelength 69.8 *μm*) TM (electric field along y direction) beam normal incident from bottom of the PI substrate (Fig. 2(a)) for example, the PI substrate dielectric constant is 3.5 and it’s thickness 100 *μm*, the V-shape copper antenna unit is as with thickness 200 nm and width of arm 1.8 *μm*. Then, to satisfy the requirement of gradient phase increase from 0 to 2π, the geometric parameters of the antennas are calculated by CST STUDIO SUITE (CST) and present as following. In detail, periodic lengths of units are p_x_ = p_y_ = 25 *μm*, the geometric parameters from 1 to 4 units are severally L_1_ = 12.1 *μm*, L_2_ = 10.5 *μm*, L_3_ = 9.8 *μm*, L_4_ = 7.5 *μm*, and *α*_1_ = 0°, *α*_2_ = 30°, *α*_3_ = *α*_4_ = 90°. The units of 5 to 8 are obtained by making a mirror symmetry for 1 to 4 units.

For this antennas metasurface shown in Fig. 2(a) with TM incidence, its scattering pattern is illustrated in Fig. 2(b), which shows that three peaks are situated at angle 0, −20 and −160 degrees, respectively. Furthermore, scattering pattern for the cross-polarized E_x_ is separately shown in Fig. 2(b) inset, in which there are two peaks near −20 and −160 degrees. By comparing Fig. 2(b) with Fig. 2(b) inset, we get the following conclusion: the V-shaped metasurface in Fig. 2(a) can produce co-polarized transmission, cross-polarized refraction and reflection at 0, −20 and −160 degrees, respectively.

In addition, the cross-polarized E_x_ field distribution along propagation direction (x-z plane) is illustrate in Fig. 2(c). In which, the cross-polarized anomalous refraction and reflection severally appear at −20 and −160 degrees in accordance with the results of Fig. 2(b).

**Figure 3:**
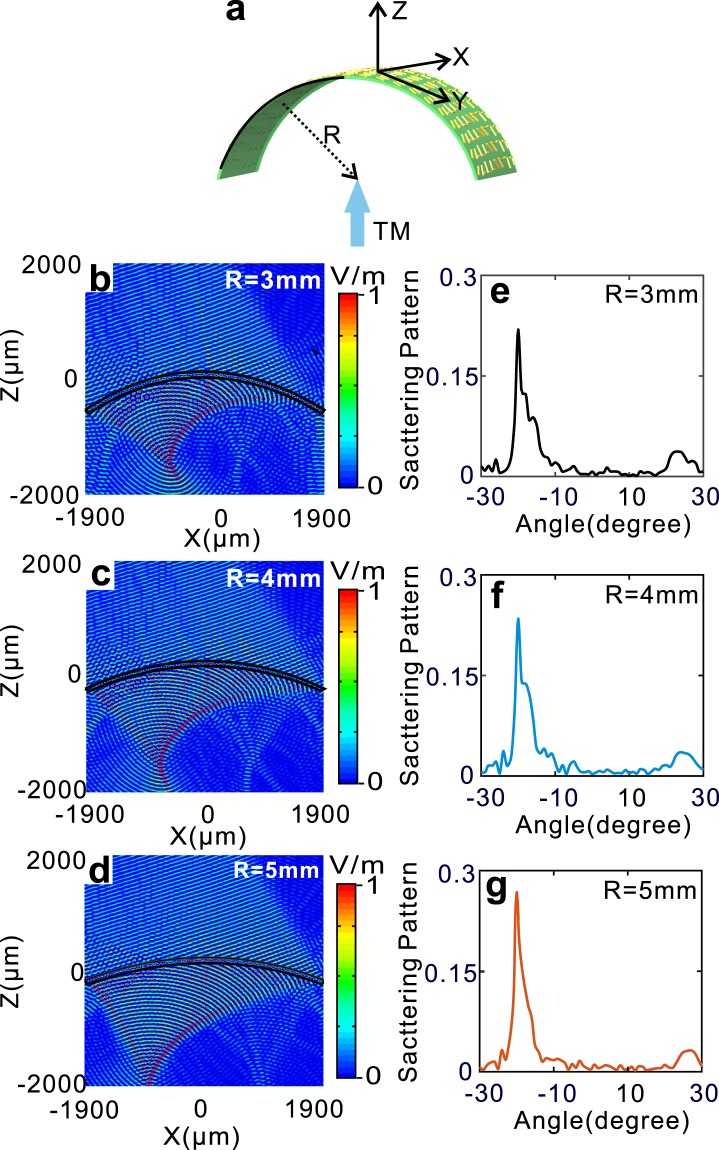
Upward bending V-shaped metasurfaces and its characteristics. (**a**) Schematic of upward bending V-shaped metasurface. (**b**–**d**) Field distributions of cross-polarized Ex for R = (**b**) 3 mm, (**c**) 4 mm, (**d**) 5 mm, respectively. (**e**–**g**) Scattering patterns of cross-polarized Ex for R = (**e**) 3 mm, (**f**) 4 mm, (**g**) 5 mm corresponding to (**b**–**d**).

To explore the optical stabilization V-shape metasurface, the anomalous refraction and reflection characteristics when the metasurface object is in bending status are numerically studied. We note that a bending can be approximately regarded as the arrangement of successive tilting. The schematic of bending metasurface is shown in Fig. 3(a). In which, R is the radius of curvature of the metasurface and TM wave is incident from bottom of the metasurface along z axis. When upward bending R are 3 mm, 4 mm, 5 mm, the cross-polarized E_x_ field distributions are severally illustrated in Fig. 3(b–d). From Fig. 3(b–d), it can be seen that show that both anomalous refraction and reflection are obtained. However, the reflection field forms converging, and for R = 3 mm, 4 mm, 5 mm, the converging points are severally near (−577, 1565), (−746, 1738) and (−811, 2172) of (x, z). On the other hand, the refraction are almost not affected, and for R = 3 mm, 4 mm, 5 mm, the scattering patterns of the anomalous refraction in Fig. 3(b–d) are correspondingly illustrated in Fig. 3(e–g) which show that the anomalous angle are all near −20 degrees. The above results are in good accordance with the former prediction from Eqs (3, 4), which imply that anomalous reflection is sensitive to the tilting rotations and the anomalous refraction only be insignificantly affected by the tilting rotation angle *α*.

**Figure 4:**
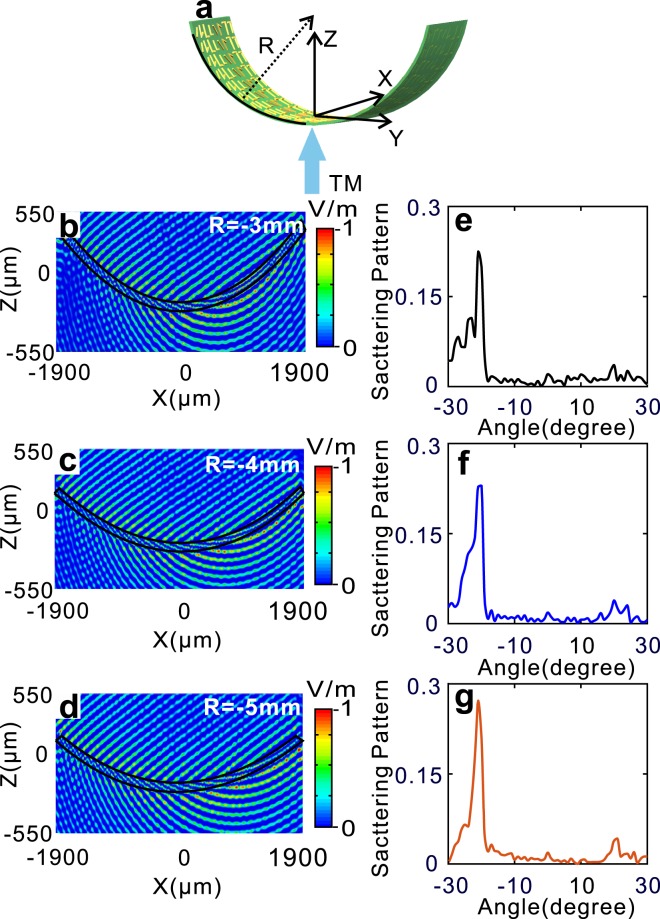
Downward bending V-shaped metasurfaces and its characteristics. (**a**) Schematic of downward bending V-shaped metasurface. (**b**–**d**) Field distributions of cross-polarized Ex for R = (**b**) −3 mm, (**c**) −4 mm, (**d**) −5 mm, respectively. (**e**–**g**) Scattering patterns of cross-polarized Ex for R = (**e**) −3 mm, (**f**) −4 mm, (**g**) −5 mm corresponding to (**b**–**d**).

For the downward bending, the shematic is shown in Fig. 4(a). When R = −3 mm, −4 mm, −5 mm, the cross-polarized Ex field distributions are severally displayed in Fig. 4(b–d), respectively, and the scattering patterns of anomalous refraction are correspondinglly illustrated in Fig. 4(e–g). Similar to the upward bending, the angle of anomalous refraction are all near −20 degree. Nonetheless, unlike the upward bending, the reflection field for the downword bending is diverging.

### Sample fabrication and THz measurements

**Figure 5:**
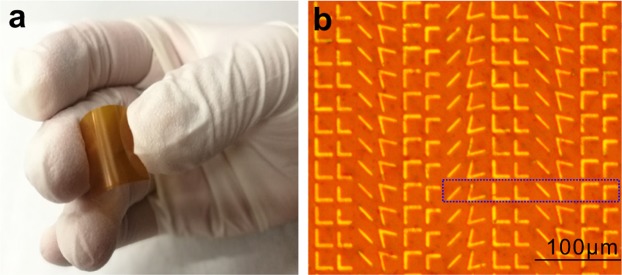
Sample fabrication. (**a**) Sample of the V-shaped copper antennas metasurface. (**b**) Part microscopic image of the sample.

**Figure 6:**
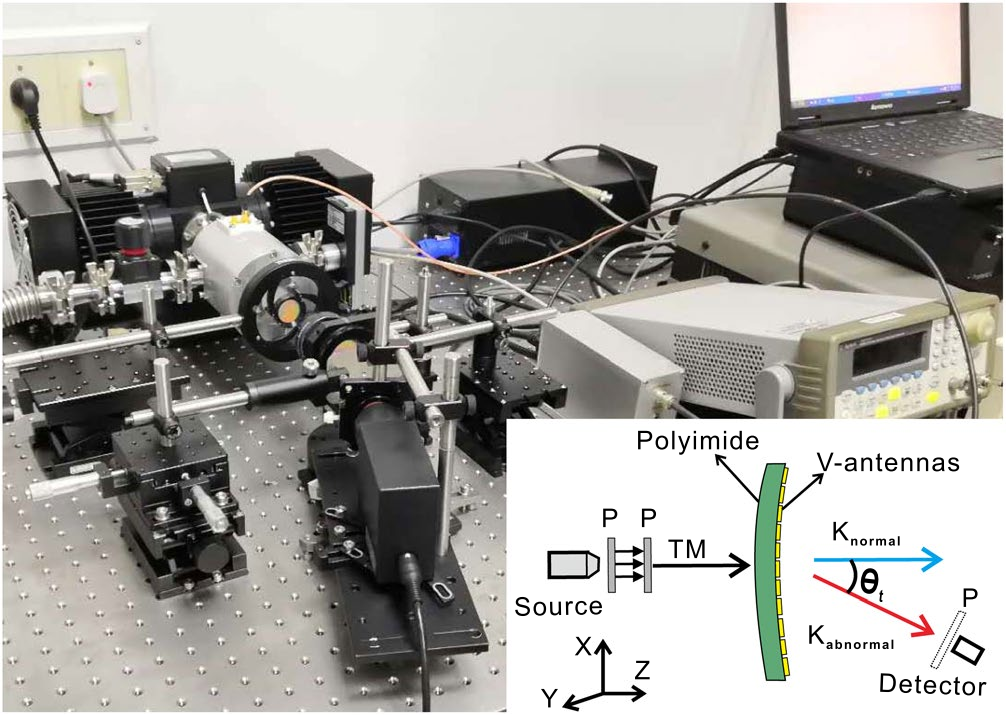
Experimental instruments of THz measurement setup with schematic diagram shown in inset.

To experimentally confirm our design idea of the bending metasurface, we fabricated V-shaped copper antennas array on flexible PI film by conventional UV lithography. In Fig. 5(a,b), the sample and the microscopic image are shown. The schematic diagram and the measurement setup of experimental instruments for sample, as shown in Fig. 6. We chose a quantum cascade laser (QCL) as the source to generate incident light at a frequency of 4.3 THz. The direction of polarization of the incident light is along the y-direction and is focused by a parabolic mirror. Two terahertz polarizers are placed sequentially in front of the sample, which is caught by two movable holders and different R are provided by tuning the distance of the holders. The polarized direction of the first polarizer is at 45 degree to x axis. The second polarizer’s polarized direction is along y axis to offer TM incidence. We chose the Golay Cell terahertz detector to receive the signal, which is 25 cm from the sample. It is mounted on a rotating table and can be rotated from −30 degrees to 30 degrees on the x-y plane to detect transmission.

### Measurement results

**Figure 7:**
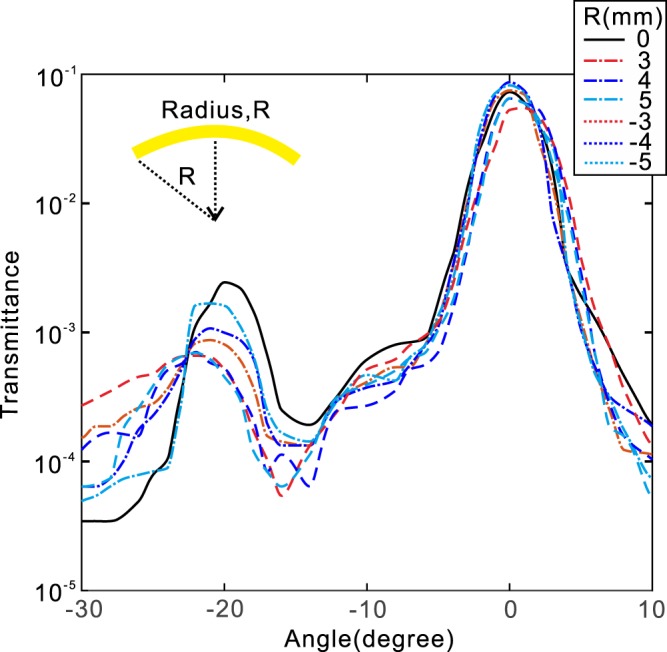
Measurement results of transmissions for bending R = 0 mm, 3 mm, 4 mm, 5 mm, −3 mm, −4 mm, −5 mm, respectively.

The experimental results of transmissions for bending R = 0 mm, 3 mm, 4 mm, 5 mm, −3 mm, −4 mm, −5 mm, are shown in Fig. 7. The results show that each curve has two peaks severally located at 0 angle and around −20 degree. Since the former numerical calculation has demonstrated that the co-polarized transmission is at angle 0 and the cross-polarized refraction at angle 20, these experimental results agree very well wtih the numerical predictions. Particularly, the refractions are almost all located near −20 degree for R = 0 mm, 3 mm, 4 mm, 5 mm, −3 mm, −4 mm, −5 mm, verifying that the refraction direction is insignificantly affected by the deformation of geometry and lead to perfect stabilization of light propagation direction.

## Discussion

The influence of the refraction and reflection when the metasurface is artificially tilted are theoretically analyzed, and the influences for upward and downward bending metasurface for R = 0 mm, 3 mm, 4 mm, 5 mm, −3 mm, −4 mm, −5 mm are numerically and experimentally studied. The results show that the anomalous reflection is sensitive to the deformation of the geometry. However, small angle tilt almost has no influence on the propagation direction of anomalous refraction, leading to perfect beam orienting. As in real-world applications, the optical objects are affected by multiple uncertain factors including deformation, vibration, non-standard surfaces, non-perfectly planar, etc., our findings shed new light on the stabilization issue of optical functionality.

## Materials and Methods

### Sample fabrication

The fabrication procedure of V-shaped copper antennas array metasurface was achieved by typical UV lithography. First, All V-shaped structures were composed of 200 nm thick copper layer on the flexible PI film by conventional electron-beam (E-beam) evaporation. After, positive photoresist SUN-115P was spin coated onto the copper film by a spin coater at a spin speed of 4000 rpm for 30 s. This was followed by hot plate baking it at 100 °C for 1 minute, the feature of V-shape antenna arrays was transferred from mask to the photoresist surface by UV lithography. Subsequently, the film was placed in developer of SUN-238D for 4 s, and then etched the exposed copper by reactive ion etching. Subsequently, the V-shaped metallization of Cu (200 nm) was evaporated and lifed-off. In order to experimentally confirm our design idea, we made several metasurfaces by typical UV lithography, as shown in Fig. 5(a,b).
